# Association between breast nodules, anxiety, depression and metabolic risk factors in a Chinese cohort

**DOI:** 10.3389/fpsyt.2023.944354

**Published:** 2023-05-18

**Authors:** Yan Long, Wei Zhang, Maolan Zheng, Qian Xie, He Liu, Xiaotong Hu, Xuelan Zhang, Wei Huang, Xia Gao, Chunmao Jiang, Can Jiang, Dong Gao, Juan Deng

**Affiliations:** ^1^Department of Health Management, Daping Hospital, Army Medical University (Third Military Medical University), Chongqing, China; ^2^Department of Geriatric Medicine, Daping Hospital, Army Medical University (Third Military Medical University), Chongqing, China; ^3^Department of Sleep and Psychology, Daping Hospital, Army Medical University (Third Military Medical University), Chongqing, China

**Keywords:** breast nodules, anxiety, depression, risk factor, psychological stress

## Abstract

**Background:**

The relationship between anxiety, depression, and metabolic parameters and the incidence of breast nodules is unclear. This study aims to investigate the association between female breast nodules and anxiety, depression and metabolic factors.

**Methods:**

This cross-sectional study recruited 857 individuals with biological indicators and breast ultrasound data from the Daping hospital from April 2021 to February 2022. Serum samples were used to measure fasting blood glucose, uric acid, triglycerides, total cholesterol, urea nitrogen, alanine transaminase, aspartate transaminase, albumin, high-density lipoprotein, low-density lipoprotein. Self-rating anxiety scale (SAS) and self-rating depression scale (SDS) were used to assess the level of anxiety and depression.

**Result:**

The positive rate of breast nodules in women aged 40 to 49 years old was significantly higher than that of other age women. The proportion of participants aged 40–49 years old in the group with breast nodules was significantly higher than that in the group without breast nodules (34.6% vs. 16.9%, *p*<0.001). Breast nodules in postmenopausal women were significantly lower than those in premenopausal women (26.4% vs. 73.6%, *p* = 0.026). The SAS scores of women with breast nodules were higher than those of the no-nodules group (40.99 ± 8.45 vs. 38.94 ± 6.89, *p*<0.001), same as the SDS scores (41.97 ± 10.33 vs. 38.91 ± 7.60, *p* < 0.001). The number of women suffering from anxiety in the group of breast nodules was significantly higher than that in control (13.8% vs. 4.5%, *p* < 0.001), and the number of depression in the group of breast nodules was also significantly higher than that of in control (14.4% vs. 4.5%, *p* < 0.001). Women with breast nodules showed a slightly lower uric acid level than those without breast nodules (290.11 ± 65.32 vs. 301.43 ± 65.93 umol/L, *p* = 0.016). Multivariable logistic regression analysis showed that age, menopausal status, anxiety and depression status were significantly associated with the presence of breast nodules, but there was no significant difference in uric acid.

**Conclusion:**

Our findings offer insight into the occurrence of depression and anxiety in the breast nodules of Chinese women. Anxiety and depression status, age and menopausal status may be the independent risk factors for the occurrence of breast nodules.

## Introduction

Breast nodules are a three-dimensional breast lesions detected by breast ultrasound, including breast cysts, solid or mixed mass, intraductal mass and breast space-occupying lesions. In recent years, with the development of ultrasound technology, the detection rate of breast nodules has gradually increased. According to data from the Netherlands and the United States, around 3% of women’s consultations with their general practitioners (GPs) are about breast changes (1, 2). Breast nodules have seriously affected the health and quality of life of women. Breast cancer is currently the malignant tumor with the highest incidence rate and mortality among women in the world (3), and 62.1% of breast cancer related deaths occur in low and middle-income countries (4). Breast nodules may increase the risk of breast cancer to some extent. It is an effective measure to diagnose and prevent breast cancer by strengthening the early health management of breast nodules, especially those with rapid growth in volume or a high possibility of malignancy in timely surgery or biopsy (5). Therefore, the risk factors of breast nodules is essential for the prevention and treatment of breast cancer.

Depression and anxiety are two major types of mental disorders that have become prominent public health problems globally (6). Women were more likely to suffer from both depressive and anxiety disorders than men, especially during periods of hormonal fluctuation (7). It has been estimated that the prevalence of depression and anxiety was 26.0% and 12.6% among Chinese women aged 40 to 60 years, respectively (8). Previous studies have found that depression and anxiety were associated with a variety of diseases, including breast cancer (9). Meanwhile, metabolic disorders including obesity, diabetes, insulin resistance, and cholesterol metabolism also played an important role in the occurrence and development of breast cancer (10, 11). Traditional Chinese medicine has long recognized that the incidence of breast diseases may be closely related to emotional factors. However, in the early stage of breast changes, there is little research on whether women’s emotional characteristics and metabolic parameters are related to the incidence of breast nodules. This study is to explore the relationship between women’s breast nodules with anxiety, depression and metabolic risk factors.

## Methods

### Study design and sample

This research met the ethical requirements and was approved by the ethics committee of Army Medical Center of PLA, in Chongqing 400,042, China (KY2020180). We collected data in Daping Hospital from April 2021 to February 2022, in Chongqing, China. The inclusion criteria included the following: (1) Women aged 18 years and older; (2) agreed to participate in this study; (3) underwent a general medical examination, which included anthropometric data, breast ultrasound and laboratory test. The exclusion criteria included the following: (1) did not agree to participate in this study or did not understand the questions in the questionnaires; (2) severe psychosis and severe cognitive impairment; (3) previous diagnosis of chronic diseases such as respiratory, cardiovascular, digestive, endocrine, nervous system or other immune diseases; (4) history of cancer (breast or other kinds of tumor). The research instruments involved demographics, anthropometric data, laboratory tests and anxiety and depression assessment. In total, 955 women were recruited. Ninety eight women failed screening: 36 subjects did not meet inclusion criteria, 51 subjects had a history of cancer, 11 subjects had invalid questionnaires. We eventually screened a total of 857 women in this study. And all participants agreed to the study and signed an informed consent form.

### Measurement

The following information was collected from a basic questionnaire for each participant: demographics, including age, marital status, menstrual history, previous medical records of chronic diseases and cancer.

All participants underwent a detailed anthropometric evaluation including height (m), and weight (kg). All of these were measured twice during the examination, and the averages of these data were used for further analysis. Body mass index (BMI) was calculated as weight/height^2^. Venous blood samples were drawn after a fasting period of 12 h. Fasting blood glucose (FBG), uric acid (UA), triglycerides (TG), total cholesterol (TC), urea nitrogen (UN), alanine transaminase (ALT), aspartate transaminase (AST), albumin (ALB), high-density lipoprotein (HDL), low-density lipoprotein (LDL) levels were measured using an Olympus AU4500 automatic chemistry analyzer (Olympus Corporation, Tokyo, Japan).

After the basic questionnaires were collected, the subjects underwent breast ultrasound examinations. Experienced ultrasound doctors used 5–36 MHz linear probes (Philips) to perform breast ultrasound examinations and subsequent evaluation on all participants. Breast cysts, solid or mixed masses, intraductal masses and breast space-occupying lesions detected by breast ultrasound were all identified as breast nodules. The location, number, size, echo, composition, edge, shape, calcification, blood flow, catheter dilation of the nodules were recorded and graded according to the BI-RADS standard (12).

The current depression status of participants was assessed using a Chinese version of the Self-rating depression scale (SDS), which was previously validated. SDS is a self-report instrument to detect symptoms related to depression and to measure the severity of depression in the general medical outpatient population. A standard score of 53 (equal to the original raw score of 41) was used as the cut-score for Chinese clinical significance. The SDS scores were classified by the following cutoff points: 53–62 was mild depression, 63–72 was moderate depression, and greater than 73 was severe depression (13). The current anxiety status of participants was assessed using a Chinese version of the self-rating anxiety scale (SAS), which was previously validated. SAS is a self-rating scale designed to detect symptoms related to anxiety in the general medical outpatient population. A standard score of 50 (equal to the original raw score of 40) was used as the cut score for clinical significance. The SAS scores were classified by the following cutoff points: 50–59 was mild anxiety, 60–69 was moderate anxiety, and greater than 70 was severe anxiety (14). An experienced psychiatrist diagnosed the depression or anxiety status of participants.

### Statistical analysis

Data were analyzed with the SPSS Version 23.0 (IBM SPSS Statistics 23). We compared the demographic and clinical variables between people with or without breast nodules by using Mann–Whitney *U*-test for continuous variables, and chi-square test for categorical variables. We adopt Spearman to explore the correlation between the degree of anxiety or depression and breast nodules. Multivariable logistic regression analysis was used to explore potential risk factors associated with breast nodules. The model for multivariable logistic regression analysis included the following covariates: age, UA, menopausal status, anxiety and depression. The assignment of anxiety and depression was defined as (0 = NO, 1 = YES) in the final model to avoid violating the principle of excluding linearly codependent variables. Odds ratios (ORs) and corresponding 95% confidence intervals (CIs) were estimated based on the multivariable model. All tests were 2-sided, and a *p*-value < 0.05 was considered statistically significant.

## Results

### Study cohort characteristics

Characteristics of the study subjects are summarized in Table 1. Of the 955 consecutive women, 11 invalid questionnaires were excluded, 51 subjects with tumors, 31 subjects did not undergo breast ultrasound, and 5 with no age information. We eventually recruited a total of 857 women for this study. Among them, 390 (45.5%) women were detected with breast nodules, and 467 (55.5%) with no breast nodules. There were no significant differences in mean age, height, weight, and BMI between the breast nodules and the control group. But the difference in age distribution was statistically significant (*p* < 0.001). The composition of breast nodules at the age between 40 and 49 was 34.6%, significantly higher than that of other age women. The proportion of participants aged 40–49 years old in the group with breast nodules was significantly higher than that in the group without breast nodules (34.6% vs. 16.9%, *p*<0.001). Breast nodules in postmenopausal women were significantly lower than those in premenopausal women (26.4% vs. 73.6%, *p* = 0.026).

**Table 1:** Demographic characteristics of participants (Means ± SD).

Variable	Breast nodules	Breast nodules	χ2	*P*
	Present	Absent		
	*n* = 390 (45.5%)	*n* = 467 (54.5%)		
Age (years)	42.99 ± 10.95	42.88 ± 12.79		0.895^a^
<30	52 (13.3%)	83 (17.8%)	3.156	0.076
30~39	96 (24.6%)	135 (28.9%)	1.989	0.158
40~49	135 (34.6%)	79 (16.9%)	35.533	0.000
50~59	88 (22.6%)	130 (27.8%)	3.116	0.078
≥60	19 (4.9%)	40 (8.6%)	4.523	0.033
			37.306	0.000^b^
**Menopausal status**				
Postmenopausal	103 (26.4%)	156 (33.4%)		
Premenopausal	287 (73.6%)	311 (66.6%)	4.930	0.026^b^
High (cm)	158.09 ± 5.14	157.40 ± 5.47		0.061^a^
Weight (kg)	55.83. ± 7.45	55.55 ± 7.48		0.583^a^
BMI (kg/m^2^)	22.36 ± 2.93	22.44 ± 2.93		0.701^a^
TC (mmol/L)	4.70 ± 0.88	4.65 ± 0.91		0.433^a^
TG (mmol/L)	1.23 ± 0.82	1.35 ± 1.15		0.084^a^
LDL (mmol/L)	2.97 ± 0.67	2.94 ± 0.69		0.493^a^
HDL (mmol/L)	1.43 ± 0.29	1.41 ± 0.29		0.337^a^
Scr (umol/L)	55.84 ± 8.50	56.22 ± 10.0		0.575^a^
UN (mmol/L)	4.87 ± 2.95	4.79 ± 1.45		0.574^a^
ALT (mmol/L)	19.66 ± 18.58	18.57 ± 18.60		0.413^a^
AST (mmol/L)	22.57 ± 7.78	22.81 ± 13.07		0.767^a^
TBIL (mmol/L)	13.62 ± 5.20	13.85 ± 4.97		0.549^a^
ALB (g/L)	45.60 ± 3.80	45.44 ± 2.23		0.470^a^
UA (umol/L)	290.11 ± 65.32	301.43 ± 65.93		0.016^a^
FG (mmol/L)	4.77 ± 0.88	4.88 ± 1.24		0.168^a^

BMI, Body Mass Index; TC, Total Cholesterol; TG, Triglyceride; LDL, Low Density Lipoprotein; HDL, High Density Lipoprotein; Scr, Serum creatinine; UN, Urea Nitrogen; ALT, Alanine Amino transferase; AST, Aspartate Amino transferase; TBIL, Total Bilirubin; ALB, Albumin; UA, Uric Acid; FG, Fasting Blood Glucose. ^a^Mann–Whitney *U*-test. ^b^Chi-square test.

### Metabolic characteristics in women with breast nodules

In Table 1. Women with breast nodules showed a slightly lower UA level compared with women without breast nodules (290.11 ± 65.32 vs. 301.43 ± 65.93 umol/L, *p* = 0.030). Except for serum uric acid, there was no significant difference in other metabolic parameters such as fasting blood glucose, triglycerides, total cholesterol, urea nitrogen, alanine transaminase, aspartate transaminase, albumin, high-density lipoprotein, low-density lipoprotein levels.

### Correlation analysis of breast nodules, anxiety and depression

**Figure 1:**
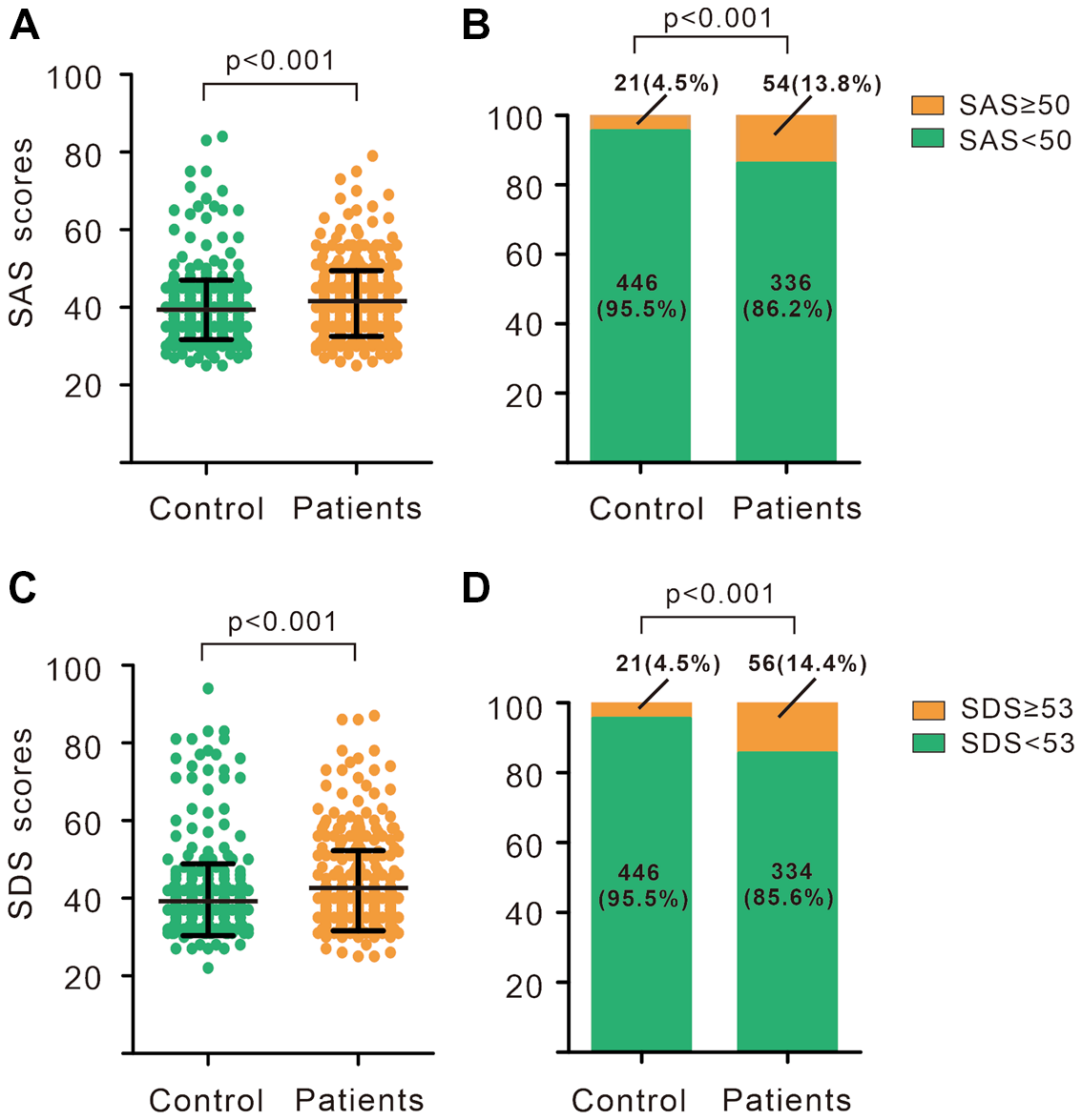
Anxiety and depression status in breast nodules patients and controls. **(A)** SAS scores of breast nodules patients and controls. **(B)** Composition of subjects with different SAS scores in breast nodules patients and controls. **(C)** SDS scores of breast nodules patients and controls. **(D)** Composition of subjects with different SDS scores in breast nodules patients and controls. **(A,C)** Wilcoxon-Mann Whitney *U*-test. **(B,D)** Chi-square test. SAS, Self-rating anxiety scale; SDS, Self-rating depression scale.

As showed in Figure 1, the SAS scores of women with breast nodules were higher than those of the no-nodules group (40.99 ± 8.45 vs. 38.94 ± 6.89, *p* < 0.001), same as the SDS scores (41.97 ± 10.33 vs. 38.91 ± 7.60, *p* < 0.001). According to the SAS scores, the number of women suffering from anxiety in the group of breast nodules was significantly higher than that in control (13.8% vs. 4.5%, *p* < 0.001), and the number of depression in the group of breast nodules was also significantly higher than that in control (14.4% vs. 4.5%, *p* < 0.001). Furthermore, the results of the Spearman correlation showed that the degree of anxiety and depression were positively correlated with breast nodules (r = 0.162, *p* < 0.001; r = 0.169, *p* < 0.001; Table 2).

**Table 2:** Spearman Correlation (r values) among the breast nodules and the degree of anxiety and depression.

Variable	Breast nodules	Control	Coefficient r	*P*
	*N*% = 390 (45.5%)	*N*% = 467 (54.5%)		
**Anxiety degree**				
No	336 (86.2%)	446 (95.5%)		
Mild	40 (10.3%)	11 (2.4%)		
Moderate	10 (2.6%)	7 (1.5%)		
Severe	4 (1.0%)	3 (0.6%)	0.162	0.000^**^
**Depression degree**				
No	334 (85.6%)	446 (95.5%)		
Mild	35 (9.0%)	9 (1.9%)		
Moderate	9 (2.3%)	4 (0.9%)		
Severe	12 (3.1%)	8 (1.7%)	0.169	0.000^**^

SAS, Self-rating anxiety scale; SDS, Self-rating depression scale.

### Factors associated with breast nodules

To explore the influence factors of breast nodules, age, anxiety, depression, menopausal status, and serum uric acid variables were included in the regression analysis. In this model, the OR of age was 1.03 (95% CI 1.01–1.05, *p* = 0.002) and menopausal status was 0.40 (95% CI 0.25–0.65, *p* < 0.001), and UA was 1.00 (95% CI 0.99–1.00, *p* = 0.038) and in women with breast nodules. There was a significant association between anxiety and depression among women with breast nodules (OR = 2.78, 95% CI 1.14–6.77, *p* = 0.024; OR = 3.98, 95% CI 1.50–10.60, *p* = 0.006; Table 3).

**Table 3:** Multivariable Logistic regression analysis of the risk factors of breast nodules.

Variables	B	SE	Wald χ2	OR (95% CI)	*P*
Age (year)	0.029	0.009	9.671	1.029(1.011–1.048)	0.002^*^
Menopausal status	−0.906	0.241	14.145	0.404(0.252–0.648)	0.000^**^
UA (umol/L)	−0.002	0.001	4.307	0.998(0.995–1.000)	0.038^*^
Anxiety	1.023	0.454	5.081	2.781(1.143–6.770)	0.024^*^
Depression	1.382	0.499	7.670	3.984(1.498–10.596)	0.006^*^

^*^Multivariate logistic regression *p* < 0.05; ^**^Multivariate logistic regression *p* < 0.001.

## Discussion

The goal of this study was to explore the relationship between women’s breast nodules with anxiety, depression and metabolic parameters. The results revealed that women with anxiety and depression were at higher risk of breast nodules. Anxiety and depression were positively correlated with breast nodules. In addition, there was a significant difference in breast nodules between postmenopausal and premenopausal. The menopausal status was negatively related to breast nodules. This effect may be stronger under the influence of estrogen (15). Meanwhile, women with breast nodules showed a slightly lower UA level than those without breast nodules, but there was no significant difference in UA in logistic regression analysis.

Breast nodules are a common problem in developing countries (16). Most breast nodules in women are benign (17). In our study, all BI-RADS classification with ultrasonography in breast nodules were equal to or less than 4a, and pathological biopsy results were lacking. Benign breast disease (BBD) is among the best-documented risk factors for breast cancer (18). Anxiety and depression are highly prevalent in the female population. The incidence of such psychological problems is known to be higher among women with malignant breast disease. However, there are few data about breast nodules or BBD. Our study showed statistically significant differences in anxiety or depression scores and levels between healthy individuals and those with ultrasound-diagnosed breast nodules, which was consistent with some previous studies in other countries (19, 20). However, there is some controversy. Fairbanks found no differences between women with BBD and healthy women in terms of the incidence of sexual dysfunction, anxiety and depression (21). One limitation of their study was the small patient sample size, which comprised 129 women. Further, we found breast nodules were positively associated with the degree of anxiety and depression. Women often experience a variety of chronic emotional stressors, including depression, anxiety, and fear, which come from marriage, family, occupation, child-rearing (22). Yet, the direct signaling network between stress pathways and the formation of breast nodules remains almost completely unknown. Indeed, chronic stress is associated with aberrantly persistent activation of the hypothalamic–pituitary–adrenal axis, leading to enhanced production of cortisol and the simultaneous elevation of catecholamines (23). Meanwhile, catecholamines may promote breast or ovarian tissue hyperplasia and canceration *in vivo* (24). Immune activity has long been established as being suppressed by anxiety and depression is considered to be responsible for promoting cancer (25, 26). Chronic stress-induced epinephrine promoted breast cancer stem-like properties via LDHA-dependent lactate dehydrogenase metabolic rewiring (27). We suspect that there may have similar mechanisms between the occurrence and development of breast nodules and emotional stressors. Anxiety and depression may be the risk factors for breast nodules. That remains to be further studied. Anxiety and depression are significant public health problems, and the lifetime prevalence of anxiety disorders for women is up to 40% (28). Anxiety and depression disorders are on the rise in all generations, both men and women, and come with substantial costs to individuals, families, and society (29). Further, it must be noted that we measured the psychological symptoms before the ultrasonic examination, most of the women showed no history of breast disease, especially breast nodules. Because the status of patients’ anxiety and depressive symptoms was not constant (30–33), further research is needed to evaluate the level of these symptoms over different periods following diagnosis. Furthermore, it must be noted that our cross-sectional study had limitations in determining a causal relationship between anxiety and depression and breast nodules.

We found that premenopausal women had a stronger correlation with breast nodules compared to postmenopausal women. From the perspective of age, the incidence of breast nodules was higher in women between 40 and 49 years old, when most were premenopausal, which was consistent with the results of several other studies (34–37). We suspect that the difference between breast nodules in age and menopause comes from the level of estrogen. The hormonal profile of BBD has generally been investigated in case–control or cross-sectional studies, frequently of modest sample size. A few studies have suggested that estrogens may be increased in BBD (38). Samoli studied 578 BBD patients and 178 healthy women in Athens, indicating that higher levels of steroid hormones, particularly estrogens, favored the development of BBD and particularly proliferative BBD (39). This may explain the lower incidence of breast nodules in postmenopausal women. After menopause, women’s breast epithelial tissue has become atrophic with changed respond to hormonal messengers (40). Serum uric acid levels are increased with aging and after menopause in females (41). Based on our study, women suffering from breast nodules had lower uric acid than controls. It has been reported that estrogenic effect could regulate the metabolism of uric acid (42). A previous study showed that people suffering from fibrocystic breast disease had a significantly lower uric acid than controls except periods of menopause and early postmenopause (15). In renal, estrogen induces fractional excretion of uric acid and higher levels of estradiol lead lower post-secretory tubular reabsorption of urate (43). However, it is still unclear whether the level of uric acid directly affects the occurrence of breast nodules. Thus, the impact of uric acid on breast nodules needs further study.

## Conclusion

In summary, our findings offered insight into the occurrence of depression and anxiety in breast nodules of Chinese women and discussed the correlation between metabolic factors, especially uric acid and breast nodules. The results indicated that women with breast nodules showed a slightly lower UA level compared with women without breast nodules. Anxiety and depression status, age and menopausal status may be the independent risk factors for the occurrence of breast nodules. Longitudinal studies to follow up patients who have breast nodules are necessary for better understanding the potential role of mental disorders in the development of breast disease.

## Data availability statement

The original contributions presented in the study are included in the article/supplementary material, further inquiries can be directed to the corresponding author.

## Ethics statement

The studies involving human participants were reviewed and approved by the Ethics Committee of Army Medical Center of PLA, in Chongqing, China (KY2020180). The patients/participants provided their written informed consent to participate in this study.

## Author contributions

JD designed the study and revised the manuscript. YL, WZ, and MZ executed the study. XZ and WH provided breast ultrasound results for all subjects. QX, XG, and ChJ collected data. CaJ and DG supervised SDS and SAS scale evaluation process. YL, WZ, and HL conducted the statistical analysis. YL and XH prepared Figure 1. YL and WZ drafted the manuscript. All authors contributed to the article and approved the submitted version.

## Funding

This work was supported by the Army Medical University Excellent Talents Foundation.

## Conflict of interest

The authors declare that the research was conducted in the absence of any commercial or financial relationships that could be construed as a potential conflict of interest.

## Publisher’s note

All claims expressed in this article are solely those of the authors and do not necessarily represent those of their affiliated organizations, or those of the publisher, the editors and the reviewers. Any product that may be evaluated in this article, or claim that may be made by its manufacturer, is not guaranteed or endorsed by the publisher.

## Abbreviations

SAS: Self-rating anxiety scale
SDS: Self-rating depression scale
GPs: General practitioners
BMI: Body mass index
FBG: Fasting blood glucose
UA: Uric acid
TG: Triglycerides
TC: Total cholesterol
HDL: High-density lipoprotein
LDL: Low-density lipoprotein
UN: Urea nitrogen
ALT: Alanine transaminase
AST: Aspartate transaminase
ALB: Albumin
ORs: Odds ratios
CIs: Confidence intervals

## References

[1] Eberl MM Phillips RL Lamberts H Okkes I Mahoney MC. Characterizing breast symptoms in family practice. Ann Fam Med. (2008) 6:528–33. doi: 10.1370/afm.905, PMID: 19001305 PMC2582463

[2] Barton MB Elmore JG Fletcher SW. Breast symptoms among women enrolled in a health maintenance organization: frequency, evaluation, and outcome. Ann Intern Med. (1999) 130:651–7. doi: 10.7326/0003-4819-130-8-199904200-00005, PMID: 10215561

[3] Bray F Ferlay J Soerjomataram I Siegel RL Torre LA Jemal A. Global cancer statistics 2018: GLOBOCAN estimates of incidence and mortality worldwide for 36 cancers in 185 countries. CA Cancer J Clin. (2018) 68:394–424. doi: 10.3322/caac.21492, PMID: 30207593

[4] Torre LA Siegel RL Ward EM Jemal A. Global Cancer incidence and mortality rates and trends—An update. Cancer Epidemiol Biomark Prev. (2016) 25:16–27. doi: 10.1158/1055-9965.EPI-15-0578, PMID: 26667886

[5] Taghipour S Banjevic D Miller AB Montgomery N Jardine AK Harvey BJ. Parameter estimates for invasive breast cancer progression in the Canadian National Breast Screening Study. Br J Cancer. (2013) 108:542–8. doi: 10.1038/bjc.2012.596, PMID: 23322203 PMC3593551

[6] Malhi GS Mann JJ. Depression. Lancet. (2018) 392:2299–312. doi: 10.1016/S0140-6736(18)31948-2 30396512

[7] Albert PR. Why is depression more prevalent in women? J Psychiatry Neurosci. (2015) 40:219–21. doi: 10.1503/jpn.150205, PMID: 26107348 PMC4478054

[8] Li RX Ma M Xiao XR Xu Y Chen XY Li B. Perimenopausal syndrome and mood disorders in perimenopause: prevalence, severity, relationships, and risk factors. Medicine. (2016) 95:e4466. doi: 10.1097/MD.0000000000004466, PMID: 27512863 PMC4985318

[9] Li T Mello-Thoms C Brennan PC. Descriptive epidemiology of breast cancer in China: incidence, mortality, survival and prevalence[J]. Breast Cancer Res Treat. (2016) 159:395–406. doi: 10.1007/s10549-016-3947-0, PMID: 27562585

[10] Kabat GC Kim MY Lee JS Ho GY Going SB Beebe-Dimmer J. Metabolic obesity phenotypes and risk of breast Cancer in postmenopausal women. Cancer Epidemiol Biomark Prev. (2017) 26:1730–5. doi: 10.1158/1055-9965.EPI-17-0495, PMID: 28939589 PMC6986334

[11] Nazih H Bard JM. Cholesterol, oxysterols and LXRs in breast Cancer pathophysiology. Int J Mol Sci. (2020) 21:1356. doi: 10.3390/ijms21041356, PMID: 32079340 PMC7072989

[12] Ellenbogen PH. BI-RADS: revised and replicated. J Am Coll Radiol. (2014) 11:2. doi: 10.1016/j.jacr.2013.11.010, PMID: 24387960

[13] Gainotti G Cianchetti C Taramelli M Tiacci C. The guided self-rating anxiety-depression scale for use in clinical psychopharmacology. Act Nerv Super. (1972) 14:49–51. PMID: 5022653

[14] Knight RG Waal-Manning HJ Spears GF. Some norms and reliability data for the state—trait anxiety inventory and the Zung self-rating depression scale. Br J Clin Psychol. (1983) 22:245–9. doi: 10.1111/j.2044-8260.1983.tb00610.x, PMID: 6640176

[15] Chen Q Xiao J Zhang P Chen L Chen X Wang S. Lower serum levels of uric acid in uterine fibroids and fibrocystic breast disease patients in Dongying City, China. Iran J Public Health. (2016) 45:596–605. PMID: 27398332 PMC4935703

[16] Singh GN Agarwal A Jain V Kumar P. Understanding and practices of Gynaecologists related to breast Cancer screening, detection, treatment and common breast diseases: a study from India. World J Surg. (2019) 43:183–91. doi: 10.1007/s00268-018-4740-5, PMID: 30051242

[17] Stachs A Stubert J Reimer T Hartmann S. Benign breast disease in women. Dtsch Arztebl Int. (2019) 116:565–74. doi: 10.3238/arztebl.2019.0565, PMID: 31554551 PMC6794703

[18] Johansson A Christakou AE Iftimi A Eriksson M Tapia J Skoog L. Characterization of benign breast diseases and association with age, hormonal factors, and family history of breast Cancer among women in Sweden. JAMA Netw Open. (2021) 4:e2114716. doi: 10.1001/jamanetworkopen.2021.14716, PMID: 34170304 PMC8233703

[19] Srivastava V Meena RK Ansari MA Kumar D Kumar A. A study of anxiety and depression in benign breast disease. J Midlife Health. (2020) 11:200. doi: 10.4103/jmh.JMH_85_20 33767560 PMC7978053

[20] Zainal NZ Ng CG Wong A Andrew B Mohd Taib NA Low SY. Prevalence of depression, trait anxiety, and social support during the diagnostic phases of breast cancer. J Taibah Univ Med Sci. (2021) 16:497–503. doi: 10.1016/j.jtumed.2021.01.013, PMID: 34408606 PMC8348272

[21] Fairbanks F Andres MP Caldeira P Abdo C Podgaec S. Sexual function, anxiety and depression in women with benign breast disease. A case-control study. Rev Assoc Med Bras. (1992) 63:876–82. doi: 10.1590/1806-9282.63.10.876 29267489

[22] Han X Li Q Wang C Li Y. The Association of Occupational Stress and Depressive Symptoms among employed persons with benign breast disease: the mediating role of psychological capital. Psychopathology. (2019) 52:205–11. doi: 10.1159/000501164, PMID: 31437833

[23] Powell ND Tarr AJ Sheridan JF. Psychosocial stress and inflammation in cancer. Brain Behav Immun. (2013) 30:S41–7. doi: 10.1016/j.bbi.2012.06.015 22790082

[24] Thaker PH Han LY Kamat AA Arevalo JM Takahashi R Lu C. Chronic stress promotes tumor growth and angiogenesis in a mouse model of ovarian carcinoma. Nat Med. (2006) 12:939–44. doi: 10.1038/nm1447, PMID: 16862152

[25] Reiche EM Nunes SO Morimoto HK. Stress, depression, the immune system, and cancer. Lancet Oncol. (2004) 5:617–25. doi: 10.1016/S1470-2045(04)01597-9 15465465

[26] Heidt T Sager HB Courties G Dutta P Iwamoto Y Zaltsman A. Chronic variable stress activates hematopoietic stem cells. Nat Med. (2014) 20:754–8. doi: 10.1038/nm.3589, PMID: 24952646 PMC4087061

[27] Cui B Luo Y Tian P Peng F Lu J Yang Y. Stress-induced epinephrine enhances lactate dehydrogenase a and promotes breast cancer stem-like cells. J Clin Invest. (2019) 129:1030–46. doi: 10.1172/JCI121685, PMID: 30688660 PMC6391112

[28] Remes O Brayne C van der Linde R Lafortune L. A systematic review of reviews on the prevalence of anxiety disorders in adult populations. Brain Behav. (2016) 6:e00497. doi: 10.1002/brb3.497, PMID: 27458547 PMC4951626

[29] Hoffman DL Dukes EM Wittchen HU. Human and economic burden of generalized anxiety disorder. Depress Anxiety. (2008) 25:72–90. doi: 10.1002/da.20257 17146763

[30] Burgess C Cornelius V Love S Graham J Richards M Ramirez A. Depression and anxiety in women with early breast cancer: five years observational cohort study. BMJ. (2005) 330:702. doi: 10.1136/bmj.38343.670868.D3, PMID: 15695497 PMC555631

[31] Osborne RH Hopper JL. Age-specific norms and determinants of anxiety and depression in 731 women with breast cancer recruited through a population-based cancer registry. Eur J Cancer. (2003) 39:755–62. doi: 10.1016/S0959-8049(02)00814-6, PMID: 12651200

[32] Vahdaninia M Omidvari S Montazeri A. What do predict anxiety and depression in breast cancer patients? A follow-up study. Soc Psychiatry Psychiatr Epidemiol. (2010) 45:355–61. doi: 10.1007/s00127-009-0068-7 19458878

[33] Keyzer-Dekker CM de Vries J van Esch L Ernst MF Nieuwenhuijzen GAP Roukema JA. Anxiety after an abnormal screening mammogram is a serious problem. Breast. (2012) 21:83–8. doi: 10.1016/j.breast.2011.08.137, PMID: 21924905

[34] Winters S Martin C Murphy D Shokar NK. Breast Cancer epidemiology, prevention, and screening. Prog Mol Biol Transl Sci. (2017) 151:1–32. doi: 10.1016/bs.pmbts.2017.07.002 29096890

[35] Nutter EL Weiss JE Marotti JD Barth RJ Jr Eliassen MS Goodrich ME. Personal history of proliferative breast disease with atypia and risk of multifocal breast cancer. Cancer. (2018) 124:1350–7. doi: 10.1002/cncr.31202, PMID: 29266172 PMC5867212

[36] Kader T Hill P Rakha EA Campbell IG Gorringe KL. Atypical ductal hyperplasia: update on diagnosis, management, and molecular landscape. Breast Cancer Res. (2018) 20:39. doi: 10.1186/s13058-018-0967-1, PMID: 29720211 PMC5932853

[37] Hartmann LC Sellers TA Frost MH Lingle WL Degnim AC Ghosh K. Benign breast disease and the risk of breast cancer. N Engl J Med. (2005) 353:229–37. doi: 10.1056/NEJMoa044383 16034008

[38] Posso M Corominas JM Serrano L Román M Torá-Rocamora I Domingo L. Biomarkers expression in benign breast diseases and risk of subsequent breast cancer: a case-control study. Cancer Med. (2017) 6:1482–9. doi: 10.1002/cam4.1080, PMID: 28470951 PMC5463091

[39] Samoli E Trichopoulos D Lagiou A Zourna P Georgila C Minaki P. The hormonal profile of benign breast disease. Br J Cancer. (2013) 108:199–204. doi: 10.1038/bjc.2012.493, PMID: 23169293 PMC3553510

[40] Olsson HL Olsson ML. The menstrual cycle and risk of breast Cancer: a review. Front Oncol. (2020) 10:21. doi: 10.3389/fonc.2020.00021 32038990 PMC6993118

[41] Zhang X Meng Q Feng J Liao H Shi R Shi D. The prevalence of hyperuricemia and its correlates in Ganzi Tibetan autonomous prefecture, Sichuan Province China. Lipids Health Dis. (2018) 17:235. doi: 10.1186/s12944-018-0882-6, PMID: 30309357 PMC6182831

[42] Yahyaoui R Esteva I Haro-Mora JJ Almaraz MC Morcillo S Rojo-Martínez G. Effect of long-term administration of cross-sex hormone therapy on serum and urinary uric acid in transsexual persons. J Clin Endocrinol Metab. (2008) 93:2230–3. doi: 10.1210/jc.2007-2467, PMID: 18349066

[43] Clegg DJ. Minireview: the year in review of estrogen regulation of metabolism. Mol Endocrinol. (2012) 26:1957–60. doi: 10.1210/me.2012-1284, PMID: 23051593 PMC3858718

